# NemaLife chip: a micropillar-based microfluidic culture device optimized for aging studies in crawling *C. elegans*

**DOI:** 10.1038/s41598-020-73002-6

**Published:** 2020-10-01

**Authors:** Mizanur Rahman, Hunter Edwards, Nikolajs Birze, Rebecca Gabrilska, Kendra P. Rumbaugh, Jerzy Blawzdziewicz, Nathaniel J. Szewczyk, Monica Driscoll, Siva A. Vanapalli

**Affiliations:** 1 Department of Chemical Engineering, Texas Tech University, Lubbock, TX 79409 USA; 2 Department of Surgery, Texas Tech University Health Sciences Center, Lubbock, TX 79409 USA; 3 Department of Mechanical Engineering, Texas Tech University, Lubbock, TX 79430 USA; 4 Ohio Musculoskeletal and Neurological Institute and Department of Biomedical Sciences, Ohio University, Athens, OH 45701 USA; 5 Department of Molecular Biology and Biochemistry, Rutgers University, Piscataway, NJ 08854 USA

**Keywords:** Caenorhabditis elegans, Lab-on-a-chip, Ageing

## Abstract

In this study, we report a microfluidic device for the whole-life culture of the nematode *Caenorhabditis elegans* that allows the scoring of animal survival and health measures. This device referred to as the NemaLife chip features: (1) an optimized micropillar arena in which animals can crawl, (2) sieve channels that separate progeny and prevent the loss of adults from the arena during culture maintenance, and (3) ports that allow rapid accessibility for feeding the adult-only population and introducing reagents as needed. The pillar arena geometry was optimized to accommodate the growing body size during culture and emulate the body gait and locomotion of animals reared on agar. Likewise, feeding protocols were optimized to recapitulate longevity outcomes typical of standard plate growth. Key benefits of the NemaLife Chip include eliminating the need to perform repeated manual transfers of adults during survival assays, negating the need for progeny-blocking chemical interventions, and avoiding the swim-induced stress across lifespan in animals reared in liquid. We also show that the culture of animals in pillar-less microfluidic chambers reduces lifespan and introduces physiological stress by increasing the occurrence of age-related vulval integrity disorder. We validated our pillar-based device with longevity analyses of classical aging mutants (*daf-2*, *age-1*, *eat-2,* and *daf-16*) and animals subjected to RNAi knockdown of age-related genes (*age-1* and *daf-16*). We also showed that healthspan measures such as pharyngeal pumping and tap-induced stimulated reversals can be scored across the lifespan in the NemaLife chip. Overall, the capacity to generate reliable lifespan and physiological data underscores the potential of the NemaLife chip to accelerate healthspan and lifespan investigations in *C. elegans*.

## Introduction

Aging is a significant risk factor for a broad range of diseases including neurodegenerative disorders, diabetes and cancer^1–5^. With the growing aging population, the socioeconomic burden attributed with age-associated diseases is staggering and development of therapies that promote healthy aging is imperative. *C. elegans* is a powerful model organism for aging investigations with a short lifespan (3–5 weeks), genetic similarity with humans (~ 38% orthologs^6^) and conserved signaling pathways^7^. Additionally, a fully mapped genome^8^ and genetic plasticity^9,10^ makes C. *elegans* an attractive tool for aging studies. Advances in fluorescent microscopy^11^ and genomic technology (RNAi, CRISPR)^12,13^ have further expanded the number of possible ways in which C. *elegans* can be used to study healthy aging.

Lifespan analysis has become a widely used method for evaluating the effects of various genes, proteins, and drug compounds on aging and age-associated diseases. In general, lifespan assays are carried out with *C. elegans* reared on agar plates containing nematode growth media (NGM). During the reproductive phase, adults must be manually transferred to new plates to separate progeny from the original sample. These animal transfers significantly increase the effort and time required for conducting lifespan assays. To reduce the work costs of manual transfers, many labs utilize a strong progeny-blocking drug (2′-deoxy-5-fluorouridine, FUdR) to maintain an adult-only population^14–16^. An alternative to this approach is the use of sterile mutants^17–20^.

The simplicity of using FUdR or sterile mutants has led to new technologies for large-scale lifespan analysis in *C. elegans*. A technology known as Lifespan Machine (LSM), allows for the rapid analysis of a population of thousands of animals grown on agar supplemented with FUdR using automatic capture of sequential images to score animal death and determine lifespan^21^. The LSM technology has provided insights into temporal scaling of ageing dynamics^21,22^ and helped identify chemical compounds with robust longevity effects^23^. Similarly, WorMotel technology facilitates longitudinal analysis of individuals in agar-filled microfabricated well plates^24^. In a recent addition, WormBot technology employed robotic platform to image and measure lifespan of animals cultured on agar surface in a 24-well plate^25^.

Although powerful, LSM, WorMotel, and WormBot technologies have some limitations. The useof FUdR is undesirable as FuDR has been shown to activate stress response pathways^26,27^, alter body size^14^, increase superoxide dismutase levels^28^, increase fat accumulation^29^ and alter the survival of worms with specific genetic backgrounds^27,29,30^. Moreover, the timing of FUdR treatment is critical, such that premature supplementation of FUdR results in abnormalities such as a high percentage of vulval protrusion, also known as age-related vulval integrity disorder (AVID)^14,15^. Another issue with the LSM and WorMotel technologies is the difficulty in studying the effect of temporal changes in environment on lifespan since these require transferring of animals to new plates. Such temporal manipulations have been central to studies of dietary restriction^31^ and functional cognitive aging^32^. In addition, uptake of drugs by animals maintained on plates is an ongoing challenge^33^.

In recent years, microfluidic approaches have begun to address the limitations of agar-based lifespan assays^34–38^. Several key advantages of using PDMS-based microfluidics include (1) excellent permeability to oxygen and carbon dioxide, enabling animals to experience natural atmospheric conditions^39^; (2) size-based separation of animals using on-chip filters^34–36^, eliminating the need to prevent or reduce progeny production; (3) precise temporal control of culture environment via addition or removal of reagents^35,36^; (4) overall reduction in the number of censored worms; and (5) optical transparency of devices to enable bright field and fluorescence imaging.

Significant advantages of microfluidics has led to the development of lifespan-devices for measuring survival curves^34–36,38^. However, a major drawback of prior devices is that *C. elegans* swims in the engineered chambers, similar to liquid cultures in multiwell plates. Physiologically, swimming in liquid culture is more energetically demanding than crawling^40,41^. In liquid environments, *C. elegans* can exhibit swimming and quiescent states^42^, making lifespan outcomes in liquid culture difficult to interpret. Also, housing worms in liquid culture for a significant portion of their lifespan has been shown to induce gene expression changes^40,43^ and oxidative stress^40^. Taken together, these findings suggest that conducting whole-life studies in microfluidic chambers where animals swim may not be representative of the physiology that crawling animals experience on plates^40,41,44^. In addition, measures such as pharyngeal pumping which can be predictive of healthspan^45–47^ are difficult to score in swimming animals.

Given the importance of mimicking plate-like behaviors in microfluidic devices, several studies have incorporated micropillars in microfluidic chambers and showed that the pillar arrangement can be configured to achieve locomotory gait similar to that on agar plates^48–52^. Additional studies have used these microstructured devices to study chemotactic behaviors^50^, separate different larval-stage animals^53^ and perform stress assays^54^. Thus, micropillar devices are well suited to induce plate-like crawling behaviors; however, to date, whole-life culturing of *C. elegans* in micropillar environments has not been demonstrated and their suitability for measuring survival curves remains to be ascertained.

Here we report an optimized microfluidic device, termed NemaLife Chip, for whole-life studies in *C. elegans* that addresses the limitations of agar-based approaches and microfluidic swim-chambers. The device has sieve channels to retain adults but enable progeny removal by fluid flow. Since previous micropillar devices have not been configured for life-long culture and survival outcomes, we optimize food concentrations and feeding frequency. Importantly, we show that whole-life culturing of *C. elegans* in chambers without pillars induces deleterious health effects. Furthermore, we show that the use of a micropillar housing arena increases the efficiency of scoring various health span metrics, including pharyngeal pumping and locomotory phenotypes. Validation of NemaLife chip through the analysis of established aging mutants, RNAi studies, and various culture conditions demonstrates that this new microfluidic device provides a simple platform with the potential to advance our fundamental understanding of genetic and environmental regulators of healthy aging.

## Results and discussion

### Optimization of the NemaLife chip design

*C. elegans* lifespan measurement in a microfluidic environment requires mimicking plate-like behaviors with the capacity to remove progeny. Prior micropillar devices^48–50^ provide some guidance on construction of environments that can recapitulate plate-like crawling behavior, while lifespan devices with swim chambers^35,36^ offer some insight into the design of sieve channels. At the same time, configuring micropillar devices for life-long culture with progeny removal and survival analysis requires new design considerations and optimal culture conditions. In this section, we discuss the device design considerations and the optimal micropillar geometry for scoring lifespan and healthspan measures. We then present optimization of cultured conditions in the next section “Optimization of worm culture conditions”.

#### Basic device design

**Figure 1:**
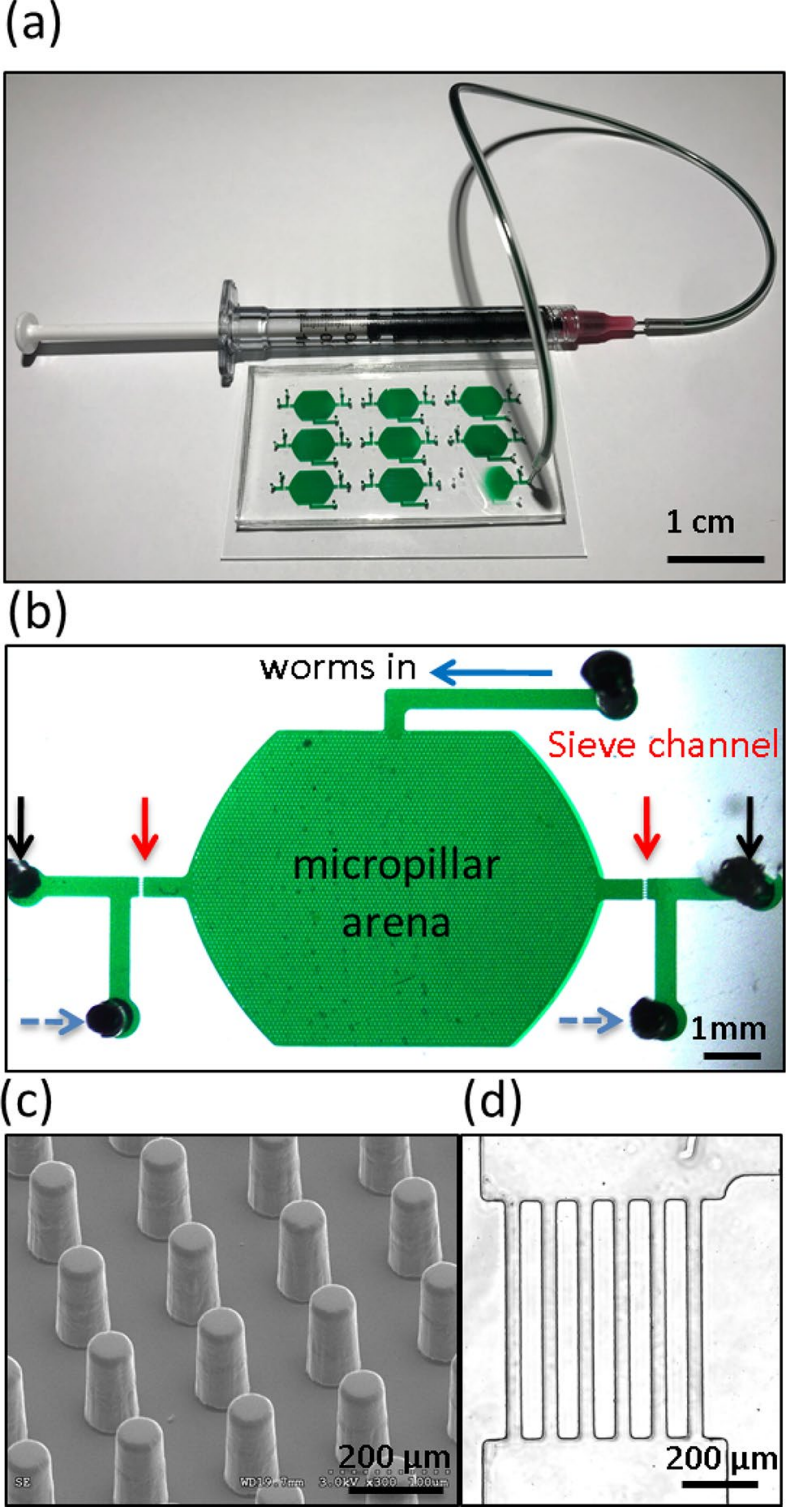
Basic design and description of the NemaLife chip. (**a**) A 9-chamber microfluidic device for lifelong studies of crawling *C. elegans*. Fluid manipulation is performed with 1-mL syringes connected to the ports in the device. Scale bar 1 cm. (**b**) Design and features of the NemaLife device filled with green dye. Habitat arena is composed of micropillars. Channel at the top (blue arrow) is the worm loading port, the two red arrows on both sides of the device identify the sieve channel that retains worms, two black arrows indicate the reagent exchange ports and the two dashed blue arrows are for purging air pockets. Scale bar 1 mm. (**c**) An enlarged view of the micropillars and their lattice arrangement. Scale bar 200 µm. (**d**) The sieve channels consist of rectangular barriers 750 µm × 75 µm separated by a gap of 25–30 µm. Scale bar 200 µm.

We designed the NemaLife culture device (Fig. 1a–d) with design objectives that feature: (1) a micropillar arena that can accommodate the growing body size as young adults are cultured and can enable animals to maintain a crawling gait throughout life; (2) optimal pillar spacing and sieve channel design that allow effective removal of progeny while retaining adults; (3) ports for introducing animals at the beginning of the experiment, washing and feeding animals, and venting air pockets that can form at the inlet during the several weeks that animals are cultured; (4) arena size for housing a population that will be easy to image and score manually while maintaining large enough sample size for meaningful statistical analysis.

These criteria were achieved by designing a worm habitat chamber that contains a micropillar lattice (Fig. 1b, c) that allows worms to crawl. Animals are introduced from the worm loading port (blue arrow in Fig. 1b) at the beginning of the lifespan experiment, and this worm loading port is then subsequently sealed with a pin. The inlet port (black arrows in Fig. 1b) is used to wash progeny and introduce *E. coli* solution to feed the animals. Sieve channels (Fig. 1d) on the sides of the habitat chamber (see red arrows in Fig. 1b), prevent young adults from escaping the arena while allowing efficient passage of eggs, larval-stage animals (L1, L2) and bacterial debris. Adjacent to the sieve channels, two side-ports (see blue dashed arrows in Fig. 1b) act as vents to purge of air pockets that can develop at the inlet of the device during overnight storage in the incubator. During washing/feeding the fluid first passes through the vent port, forcing the air out before the introduced fluid enters the pillar arena. Fluid manipulation is performed manually using hand-held syringes, an aspect of design that we felt would make the device more accessible to use in a wide range of laboratories than would a complex pumping set-up.

#### Micropillar arena design

Worm habitat chambers are designed to have an approximate footprint of  ≈ 60 mm^2^ for a population size of 10–15 animals (4–6 mm^2^ per animal), compared to an average footprint of 2828 mm^2^ in standard studies on agar for 30–50 animals (60–90 mm^2^ per animal). We were able to accommodate nine of these chambers on a 50 × 75 mm^2^ glass slide (Fig. 1a) that could be used to generate survival data on ≈ 100 animals.

The geometry of the micropillar lattice in the NemaLife chamber is crucial for successful measurement of lifespan of *C. elegans*. The lattice structure must accommodate changes in body size during reproduction and aging, maintain the natural crawling gait of *C. elegans*, and allow non-invasive removal of progeny while retaining the sample population. To identify the optimal micropillar lattice that simultaneously meets these requirements, we fabricated devices with square arrangement of pillars with different pillar diameter (*a*) and edge-edge gap (*s*). The nominal dimensions we tested are: Device I, *a* = 40 μm, *s* = 60 μm; Device II, *a* = 50 μm, *s* = 80 μm and Device III, *a* = 60 μm, *s* = 100 μm. The measured dimensions of the three pillar devices are reported in Table S1. During the greatest period of growth, C. *elegans* body diameter varies from ~ 50 to 100 µm and length varies from ~ 900 to 1500 µm (Fig. S1). Thus, Device I provided the tightest, and Device III the leanest, confinement for the animals during the lifespan measurement.

In all the devices, the pillars had a uniform height of ≈ 75 μm and a clearance from the floor of the habitat chamber of approximately ≈ 25 μm, allowing the pillars to be moved aside by the animal to adjust gait and accommodate changes in body size. The gap between the pillars and the clearance between the pillar tip and the floor also helps in size-based separation of the adults from the larvae and eggs. An adult’s body diameter is much larger than the clearance and the animal interacts with approximately 10–16 pillars along its body, which prevents the animal from being washed away during fluid flow. Specifically, animals maintain their posture and crawl normally using the pillar support while the progeny, eggs and other smaller particles are removed via fluid flow.

#### Sieve channel design

Our optimized sieve channels have a width of 25–30 μm, length of 750 μm, and are separated from each other by 75 μm × 750 μm rectangular blocks. We designed the length of the rectangular blocks long enough so that the animal cannot generate natural waves and propagate through the channels. In addition, we found that the placement of the sieve channel relative to the pillars is important as animals tend to use the pillars to generate thrust to force themselves through the channel. Placing the sieve channels at a distance of 1000 μm away from the nearest pillar mitigated this behavior and allowed the animal to quickly reverse back to the arena when it approached the sieve channel area.

#### Selection of the optimal micropillar geometry based on locomotion and lifespan outcomes

**Figure 2:**
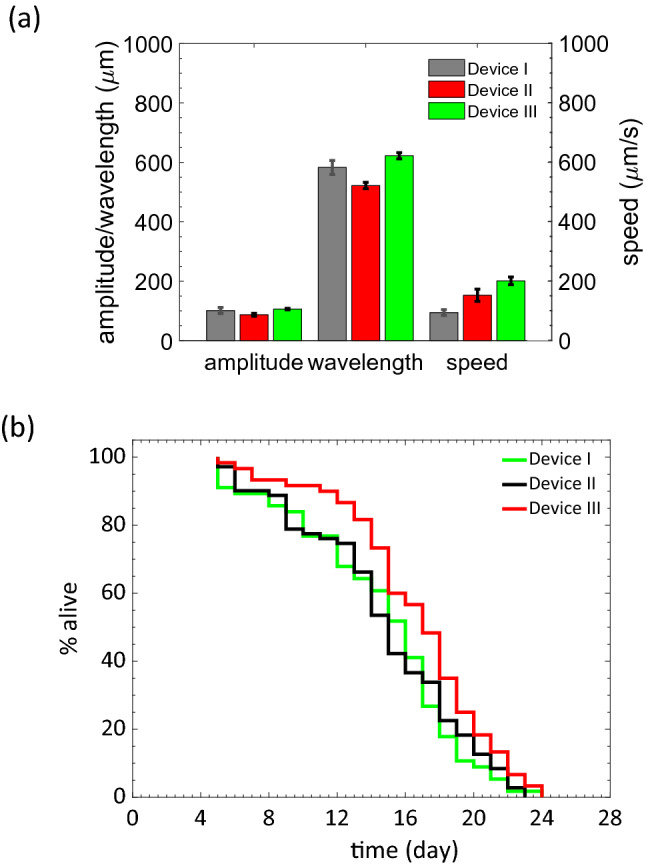
Influence of micropillar geometry on animal locomotion and lifespan. (**a**) Crawling amplitude, wavelength, and speed of day 4 animals as pillar spacing was changed within the three devices (n = 10). Pillar diameter and edge-edge gap for Devices I, II and III are: 40 μm, 60 μm; 50 μm, 80 μm; 60 μm, 100 μm. Older adults are most constrained in Device I and are not constrained in Device III. (**b**) Wild type *C. elegans* lifespan as a function of confinement (n = 56 for Device-I, n = 71 for Device-II and n = 60 for Device-III). *p* value for lifespan curve between Device-I and II is 0.99 and p-value for lifespan curve between Device-I and III is 0.026 (Log-rank (Mantel-Cox) test, N = 2 repeat trials. Data is shown for one trial.

We initially measured the crawling gait in terms of wavelength, amplitude and crawling speed in the three devices for day 4 animals (from hatching). The amplitude and wavelength corresponding to the worm undulatory motion in the three devices were similar (Fig. 2a). However, the crawling speed was diminished in Devices I and II compared to Device III due to their tighter confinement.

Also of interest is the comparison of data in the test devices to that of animals crawling on agar^55^. We found that the amplitudes are similar between the two^52^. However, the wavelength was about 30% higher on agar. Animals do crawl in Device III with a similar speed to animals crawling on agar surfaces^55^. Thus, among the three devices tested, animals housed in Device III have a locomotory gait that is most similar to that observed on agar plates.

We conducted survival experiments in the test devices at the optimal feeding protocol of 100 mg/mL of *E. coli* OP 50 with animals being fed once a day (see “Optimization of worm culture conditions” section for the feeding optimization study). Figure 2b compares the survival data for the animals reared in the three devices. Devices with tighter pillar spacing resulted in a reduction of worm lifespan, possibly due to restraints in natural locomotion and stress conferred through body confinement. We found that median and maximum lifespan in Device III were most consistent with studies on agar (see “Validation of the NemaLife chip” section) We conducted all subsequent aging investigations using Device III with s = 100 µm.

#### Effect of population density on animal lifespan in the micropillar chamber

Population density in each micropillar chamber may limit food availability as well as accumulation of small molecules such as pheromones that help animals sense overcrowding^56^ and makes them enter into a non-aging dauer-stage^57^ or extends their lifespan by reducing the insulin signaling^58^.

To investigate the effect of population density, we conducted lifespan experiments in which we varied the number of animals per chamber and manipulated the micropillar chamber size to vary the population density (see Table 1). We maintained the culture in the chamber by daily washing and feeding with 100 mg/mL of *E. coli* OP50. In Table 1 we compare the median lifespan of animals experiencing population densities ranging from 4.0 to 12 mm^2^/animal. We note that population density is typically defined as number of animals per unit area, but here we chose the inverse unit to highlight the pillar area needed for chamber design. We find that the median lifespan corresponding to different population densities is not significantly different compared to our standard NemaLife chip condition of 4–6 mm^2^/animal. This data suggests that in the explored range of animal density, the NemaLife chip does not introduce deleterious effect on animal lifespan.

**Table 1:** Influence of population density and number of animals per chamber on lifespan of wild-type animals.

Area (mm^2^) per animal	Animals per chamber	Number of trials	Animals scored per trial	Median lifespan, day	Statistical significance
4.0–6.0	10–15	5	86–100	13.6 ± 0.56	–
7.0	1	4	59–91	14.2 ± 0.75	n.s
10–12	60–70	2	60–70	13.5 ± 0.71	n.s

Statistical comparison of survival curves were made with respect to our standard assay condition (highlighted in bold) using log-rank test. *p* value of > 0.05 corresponds to not significant (n.s.).

Additionally, Table 1 shows the lifespan data when an individual animal is housed in the chamber which prevents transmission of the chemical/hormonal signals due to physical isolation. Still, we find that lifespan data from the single-animal chamber is comparable to that of our standard NemaLife chip suggesting that in the explored range of population densities, animal crowding does not have a major influence on lifespan. Recognizing that the population density in the chip is much higher than on plates, we believe that daily washing and supply of fresh food may be the reason that animal density may not be as influential in the chip as it is on plates.

Although the different population densities in the chips show comparable lifespan, the live/dead scoring time increases significantly as the population and chamber size increases. Alternatively, having too few animals per chamber will require more chambers to reach an adequate population size for a particular study. We chose to use 10–15 animals per chamber as an optimal trade-off between ease of scoring and achieving a sample size of ≈ 100 animals per assay condition.

### Optimization of worm culture conditions

We established culture maintenance protocols to achieve robust and reproducible lifespan data while increasing the overall efficiency of conducting survival analyses. Initially, we focused on identifying the optimal washing conditions necessary to remove all progeny. To do this, we cultured reproductive adults within the NemaLife device for 24 h to allow progeny production and growth. We then tested progeny removal by delivering 200 µL aliquots of S-complete media via the loading port of the device.

**Figure 3:**
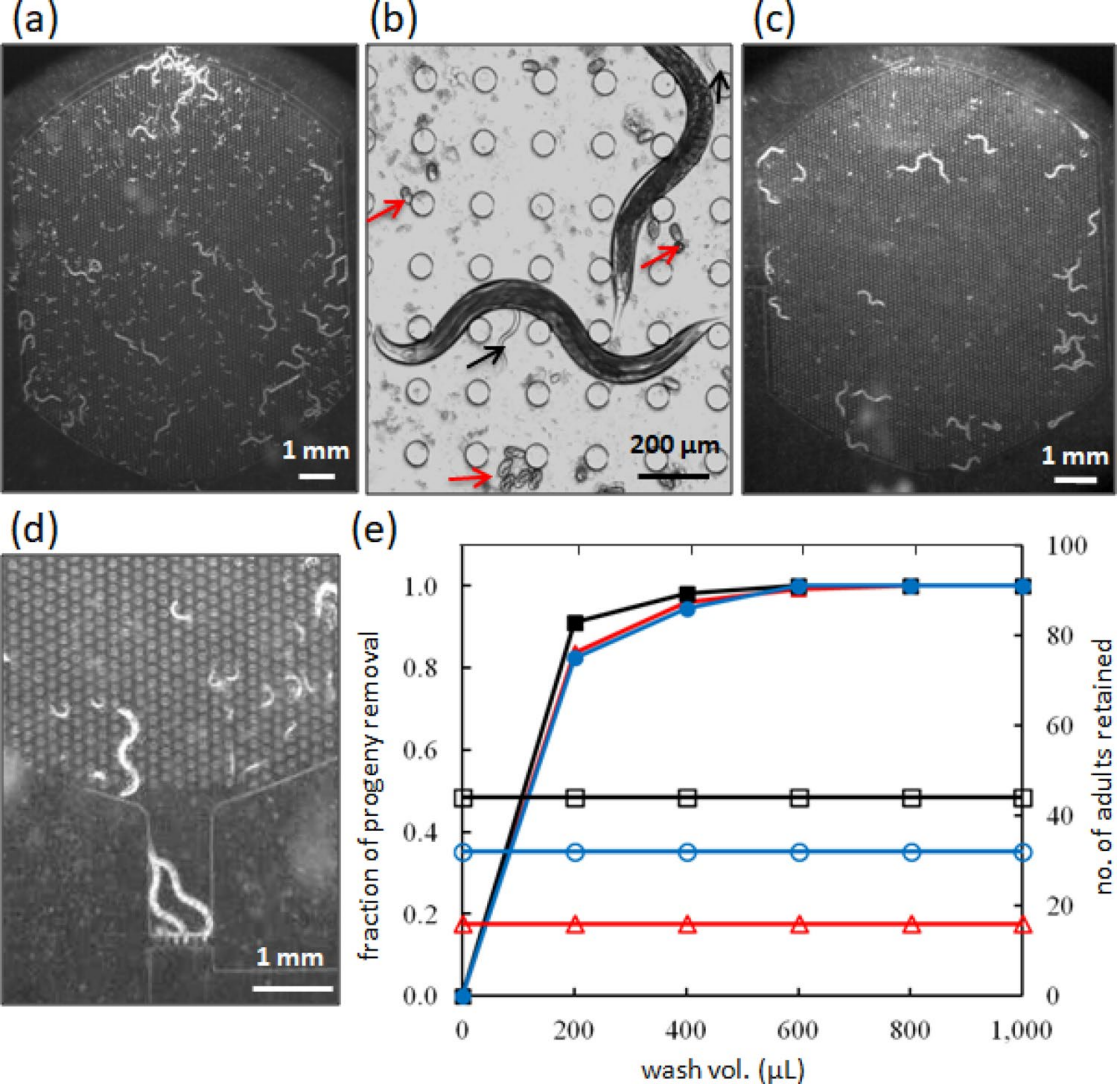
Worm culture in the NemaLife chip with capacity to remove progeny. (**a**) A typical chamber with wild-type animals and their progeny 24 h after loading. Scale bar 1 mm. To easily visualize progeny, the effect of progeny washing, the images contain about 40 animals, which is almost 3 × the standard count of 10–15 animals per arena. (**b**) Enlarged view of adult animals, progeny (black arrows) and eggs (red arrows) inside the micropillar arena. Scale bar 200 µm. (**c**) A chamber with adult-only population after removal of progeny/eggs by washing. The tiny white spots represent bacterial aggregates and not eggs. Scale bar 1 mm. (**d**) Animals at the exit are retained by the sieve channel, Scale bar 1 mm. (**e**) Effectiveness of progeny removal and adult retention in the NemaLife chip by washing the chambers with S-complete buffer. 16 (red), 32 (blue), and 44 (black) adults (day 3 after hatching) were allowed to reproduce in 3 identical units and synchronization was performed on day 4. Animals were incubated at 20 °C. Open symbols represent adults and closed symbols represent progeny. Adult retention and progeny removal from a single unit is identified with the same color and symbol. N = 3 repeat trials. The device used in efficacy trial for progeny removal has pillars of diameter 40 µm and spacing of 60 µm (Device I).

Figure 3a, b shows the habitat chamber with adults and their progeny (see SI movie 1). After washing, the retained adults are shown in Fig. 3c. In the event that the animals are already near the exit, sieve channels retain them inside the chamber (Fig. 3d, SI movie 2). We found that all adults were retained, and all progeny were effectively removed using a total wash volume of 1 mL for three different loading conditions (Fig. 3e; SI movie 2). The washing operation took approximately 90 s.

All trials were successful in removing progeny. We washed each chamber and used a stereomicroscope to determine for efficacy of progeny removal. During washing, the progeny that are washed out are eggs and L1 larvae. In case any progeny are left out accidently, these progeny can be L2 (diameter is ~ 25–35 μm) or L3 (diameter is ~ 40 μm) and can still be washed out the next day through the sieve channel of width 25–30 μm. Occasionally when bagging (internal hatching of progeny which can kill the mother) occurred, more repeated washes (2–4 mL) were necessary to remove the progeny of bagged mother or the resulting advanced larval stage progeny. We note that during the washing process, animals in the chamber respond by exhibiting faster crawling momentarily, probably due to the stimulation by fluid forces (see SI movie 2).

Next, we sought to establish a robust feeding protocol that provides the worms with an adequate supply of food. *E. coli OP50* is widely used as the standard diet for *C. elegans* cultures. Previous lifespan studies in multi-well plates^16,59^ and pillar-less microfluidic devices^34,36^ used a bacterial concentration of 10^9^–10^10^ bacterial cells per mL of S-medium and added the bacterial suspension to the microfluidic devices either continuously^36^ or once a day^34^.

In our study, we used the bacterial concentration from previous studies as a starting point to optimize the feeding protocol and evaluate the lifespan. The feeding conditions we tested include 100 mg/mL *E. coli* OP50 once a day, twice a day, or every other day, where 100 mg/mL *E. coli* OP50 was found to be equivalent to ≈ 2.4 × 10^11^ colony forming units (cfu)/mL. In addition, we also increased food by testing 200 mg/mL *E. coli* OP50 once a day. We note that the microfluidic chamber can hold ≈ 6.75 μL and ≈ 250 μL of food volume was injected into the device, indicating there was no significant food dilution occurring in the chamber.

**Figure 4:**
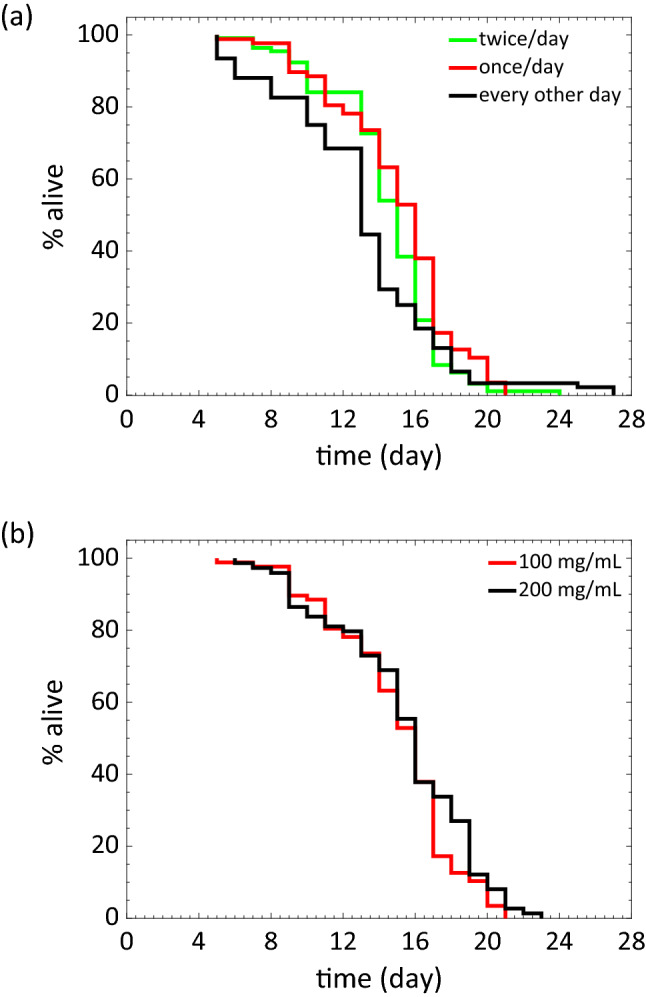
Optimization of feeding protocol for *C. elegans* lifespan assays. (**a**) Lifespan of wild type *C. elegans* for feeding frequency of twice per day (n = 112), once per day (n = 87) and every other day (n = 92). N = 2 repeat trials. Food concentration: 100 mg/mL of *E. coli* OP50 in S-complete. (**b**) Lifespan of wild type *C. elegans* for food concentration of 100 mg/mL (n = 87) and 200 mg/mL (n = 74) of *E.coli* OP50 in S-complete. N = 1 trial. Feeding frequency: once every day. Feeding animals every other day, produces a lower median (and mean) and higher maximal lifespan than feeding every day (*p* = 0.0036) or twice per day (*p *< 0.001). *p* value (once per day vs twice per day) = 0.0244.

We find that worms fed every other day at 100 mg mL^−1^ had an overall extension in maximum lifespan but a significant decrease in median lifespan as well as a high death rate during reproduction (Fig. 4a). Alternatively, we noted that feeding twice per day did not significantly alter lifespan (Fig. 4a) or age-dependent changes in body size (see SI Fig. S1). Figure 4b shows that animals fed once a day at 100 mg/mL or 200 mg/mL had similar survival curves suggesting that feeding 100 mg/mL daily is sufficient for measuring survival curves.

**Figure 5:**
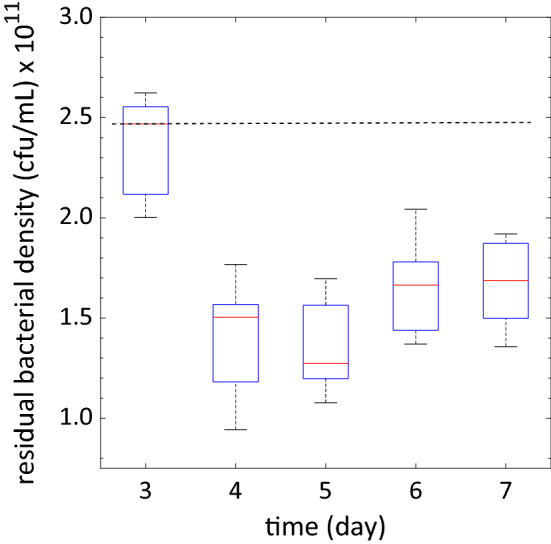
Residual food concentration after a cycle of incubation during reproductive period. Consumption of bacteria by the adult and their progeny over 24 h’ time (once per day feeding) based on measuring left-over bacteria in the device. Dashed horizontal line represents the daily food concentration, equivalent to 100 mg/mL, provided to the animals each day. Day 3 is the day of loading animals into the chip at age 60–65 h from hatching (day 1 adult). Sample size = 8 chamber/day and 9–14 animals in each chamber. Error bar is the standard error.

Next, we assessed whether the animals might become dietary restricted when supplied with a daily dose of 100 mg/mL bacterial solution. We measured the density of the bacterial solution at the end of a feeding cycle by collecting the washed fluid from each NemaLife chip. Each chip had 9–14 animals and 8 chips were used as replicates. Figure 5 shows the residual bacterial density measured daily during the reproductive period, starting from day 3 until day 7, to account for the consumption of bacteria from hatched progeny. We found that the bacterial density dropped significantly after day 3 and remained approximately the same (≈ 1.5 × 10^11^ cfu/mL) during the rest of reproductive period.

Dietary restriction has been shown to occur on plates at bacterial densities of 1 × 10^8^ cfu/mL^31^. The food levels remaining after daily feeding are substantially above the dietary restriction levels even during the peak reproductive period when adults are consuming significantly more and hatched progeny also contribute to the depletion of food sources^60^. Thus, we conclude that a diet of 100 mg/mL *E. coli* OP50, added once daily, is sufficient for whole-life culture of *C. elegans* in the NemaLife chip.

### Whole-life *C. elegans* studies in pillar-less microfluidic chambers

Previous microfluidic works focusing on lifespan measurement in *C. elegans* used chambers without pillars^34–38^. In these pillar-less microfluidic devices, animals experience swim-induced physiological stress, potentially affecting lifespan and healthspan outcomes. To clarify the potential impact of life-long swimming, we used the same overall design of NemaLife chip but without the pillars in the arena. We used the optimized washing and feeding protocol and conducted in parallel whole-life studies in the pillar-less and pillar-equipped chips. Specifically, for animals cultured in both environments, we measured survival curves and scored for the age-related vulval integrity disorder (avid) phenotype as a measure of physiological health.

**Figure 6:**
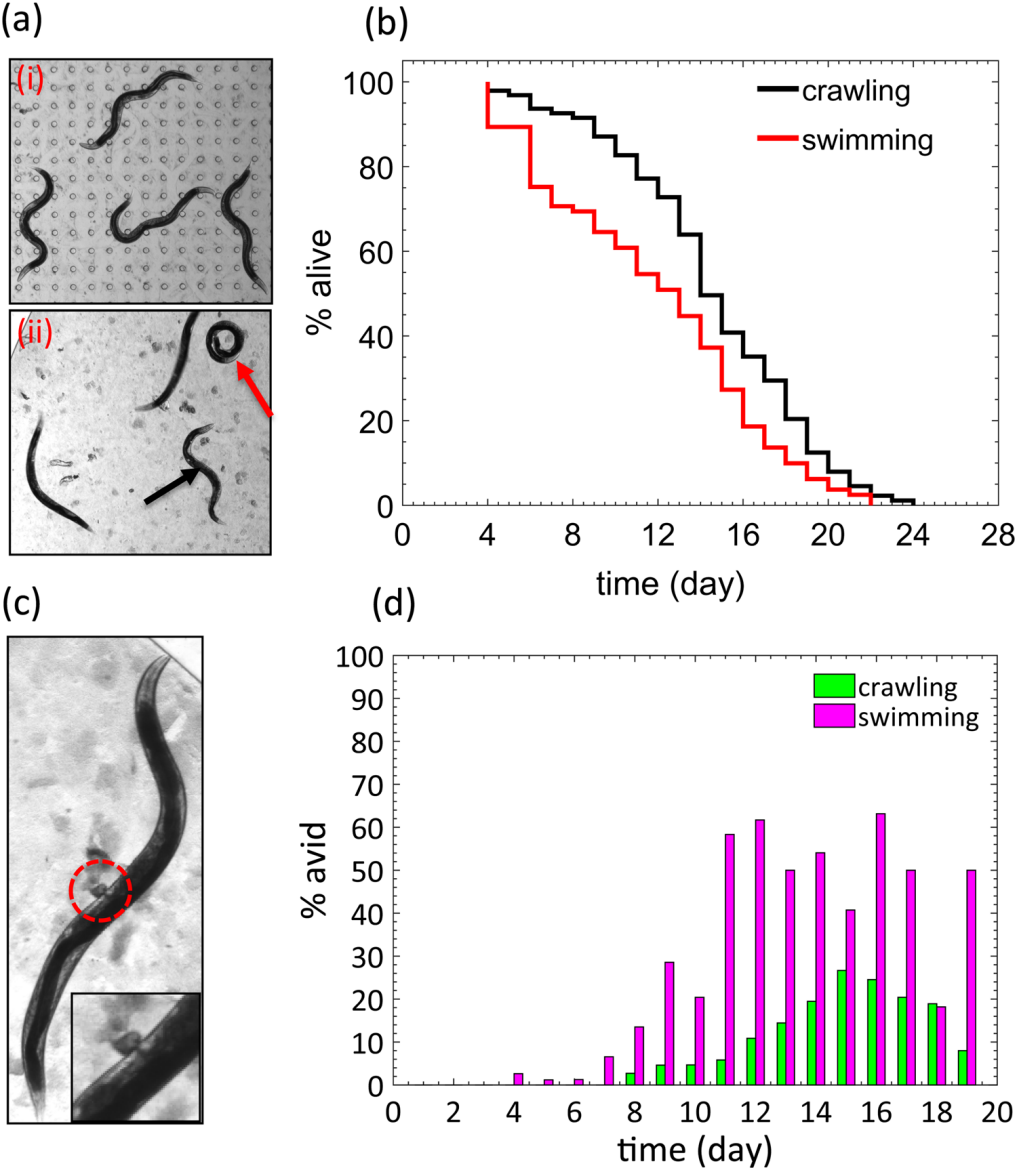
Lifespan and healthspan outcomes due to whole-life culture in pillar-less microfluidic chambers (**a**) (i) A representative snapshot of wild type *C. elegans* (day 5) crawling in a micropillar environment exhibiting typical postures. (ii) Snapshot of wild type animal swimming in the pillarless chamber. Black arrow shows a fatigued animal and red arrow shows a coiled animal. Animal age: day 5. (**b**) Lifespan of crawling versus swimming wild type *C. elegans* at 20 °C (*p* < 0.001). N = 2 trials, data is shown for one trial. Sample size in pillar-less and micropillar environment is 97 and 95 respectively. (**c**) A day 8 *C. elegans* with age-related vulval integrity disorder (avid) shown in a dashed red circle. Enlarged view is in the inset. (**d**) Percentage of animals showing avid phenotype in the pillar-laden (crawling) and pillar-less (swimming) devices.

Comparing the progeny-washing process between devices with and without pillars, we found that in the pillar-less chip, progeny removal is challenging and requires additional wash steps since the animals accumulate at the sieve channels blocking the flow of fluid (see SI movie 3). Thus, additional washes were needed to ensure removal of progeny in pillar-less chips. Another important distinction is that although animals in the pillar-equipped NemaLife chip crawled naturally (Fig. 6a, i), many animals in the pillar-less chip were found to assume a stiff (black arrow in Fig. 6a, ii) and stationary posture as well as show occasional coiling behavior (red arrow in Fig. 6a, ii). These unnatural body postures in pillar-less microfluidics chambers suggest that animals suffer from swim-induced fatigue^40,42^.

In Fig. 6b, we show the survival curves for animals in both the pillar-free and pillar equipped environments. The median lifespan of animals cultured in the pillar-less environment was 11 days compared to 14 days in the pillar environment. However, maximum lifespan was similar for the two populations. Thus, animals experiencing swim-induced physiological stress have shorter mean lifespan.

The larger death rate during early reproductive period in the pillar-less chip is due to animals showing age-related vulval integrity disorder (avid). Avid is characterized by vulval protrusion and expulsion of intestinal fluid or tissues (see Fig. 6c) which has been shown to increase with hypoxic stress, decreasing temperature, and poor reproductive health^61^. In the pillar-laden chips, we observed the first instances of avid after the reproductive period, which is consistent with studies on plates^61^. However, we find that worms cultured in the pillar-less chambers experience avid as early as day 4 and avid occurs more frequently compared to animals in the pillar-environment (Fig. 6d). In sum, the reduced lifespan and the frequent occurrence of the avid phenotype in the swim-chambers observed in the absence of pillars, suggest that the pillar environment of the NemaLife chip is crucial for mitigating stress during life-long liquid culture of *C. elegans*.

### Validation of the NemaLife chip

Next, we discuss studies that were conducted in the NemaLife chip to validate our microfluidic approach for lifespan measurement in *C. elegans*. Specifically, (1) we compared the lifespan data for animals cultured in our microfluidic devices versus those maintained on agar plates, (2) we compared the stress induced in the NemaLife chip to that on agar plates, (3) we measured lifespan of mutants with known aging pathways, and (4) we tested efficacy of RNAi interventions in the device. The lifespan data corresponding to all these conditions is included in Table S2 in the Supplementary Information.

#### Comparison of *C. elegans* lifespan in device and on agar

**Figure 7:**
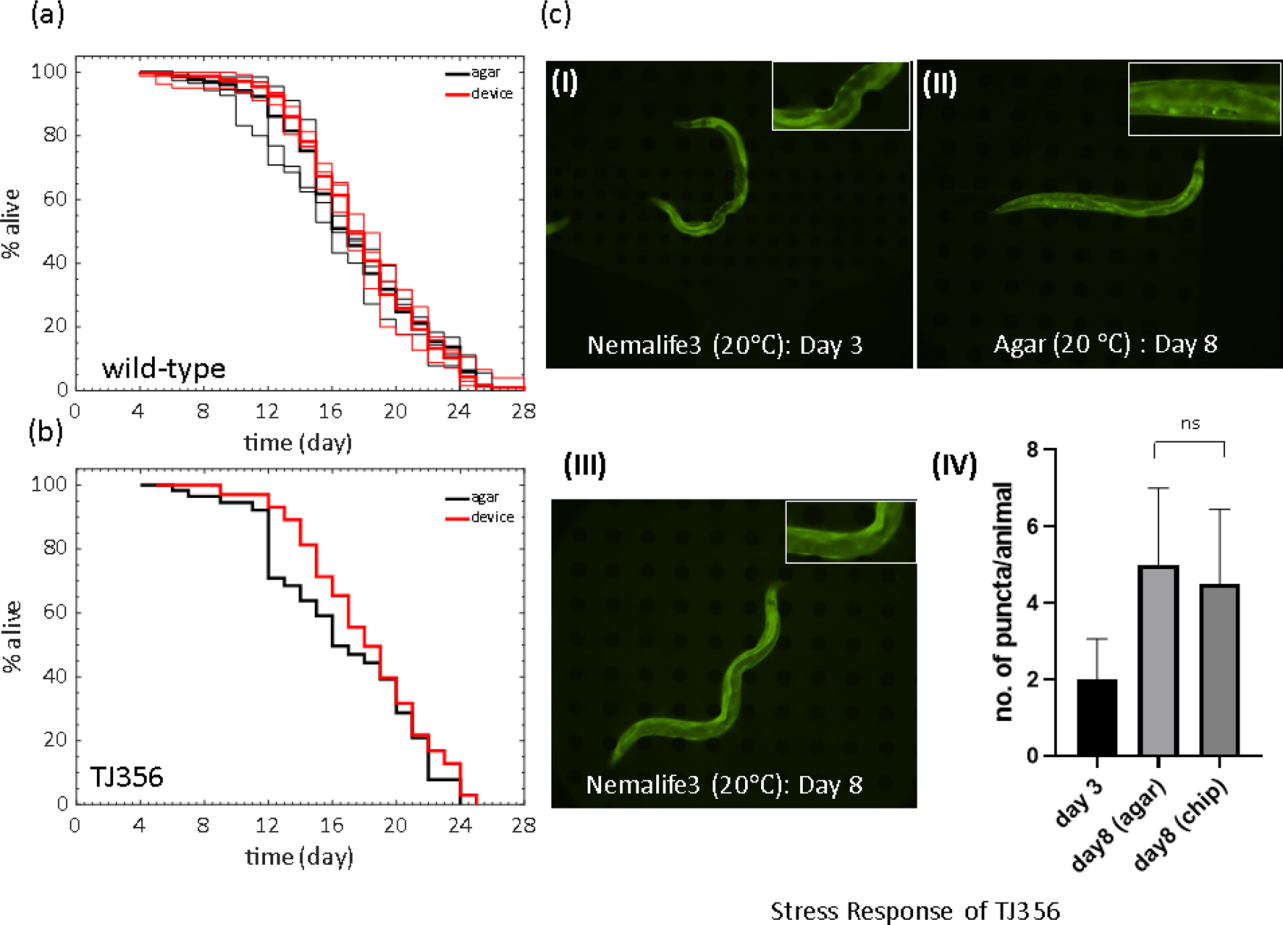
Lifespan measured in the NemaLife device is similar to that animals reared on agar plates: (**a**) Lifespan of wild type *C. elegans* evaluated on agar plates and in the microfluidic pillar device. Thin solid lines represent independent trials and the thick solid line represents the combined lifespan of the trials. Lifespan evaluated in microfluidic device is consistent with the agar plate assay (*p* = 0.11 for trial-1; *p* = 0.13 for trial-2 and *p* = 0.22 for trial-3, log rank test) at 20 °C. Sample size in agar/microfluidic device—trial-1: 109/81; trial-2: 159/135; and trial-3: 70/112. (**b**) Lifespan of a transgenic strain TJ356 with a Pdaf-16::GFP stress reporter on agar plates and in the microfluidic device (*p* = 0.16, log rank test). Sample size is 71/112 (agar/microfluidic device) at 20 °C. N = 2 trials, data is shown for one trial. (**c**) Fluorescent imaging of DAF-16::GFP nuclear localization of live worm in the microfluidic device. (i) no sign of accumulation in a day 3 (from hatching) when freshly loaded into the device from agar plate at 20 °C, (ii) and (iii) are images of 8-day old animal (from hatching) on agar plate and in microfluidic device at 20 °C respectively (Insets show zoomed-in fluorescence images), and (iv) number of visible puncta in animals cultured in agar plate (n = 5, day 8 animals) and in NemaLife chip (n = 10, day 8 animals) (*p* = 0.65, unpaired *t* test).

To evaluate if our optimized NemaLife device generates *C. elegans* lifespan data consistent with that of standard agar plate assays we conducted parallel lifespan analysis of young adults using established protocols. Figure 7a shows that housing worms in the NemaLife chip does not significantly alter lifespan (*p* > 0.11). The median and maximum lifespan from three replicates on agar were 15.67 ± 0.58 and 24.33 ± 0.58 days respectively. Likewise, the median and maximum lifespan in the microfluidic device were 16.0 ± 0.0 and 24.67 ± 2.08 days, respectively. Here, the maximum lifespan is taken as the day before the last animal of a population is dead.

Although we find that the lifespan curves are in good agreement between NemaLife chip and agar assays, we find that the NemaLife chip offers considerably less animal loss during a survival assay. On agar, we found 5–30% animal loss over the course of the assay. It is common to lose animals on agar plates due to (1) crawling up along the side wall and death from desiccation, (2) burrowing into the soft agar which precludes scoring, and (3) bagging. Animal loss due to desiccation and burrowing depends on the type of strain, sex, mutation, and intervention used, and sometimes may account for as much as 50% of the total animal population^62^. As death events due to desiccation and burrowing cannot be scored, whether agar-based lifespan is a measure of a selected subset of a population becomes a question. Moreover, loss of animals increases the number of worms needed to initiate each experiment as loss must be anticipated.

We found that the NemaLife chip eliminates the incidences of animal loss from desiccation and burrowing due to culture in an enclosed microfluidic chamber. We observed a 0–6% animal loss, which could be attributed to washing mistakes (human error of not plugging the loading port, which lacks a sieve channel, or of excess pressure application for fluid flow such that animals close to the sieve channel squeeze out). Animal loss/censoring from bagging was 1–6% in the NemaLife chip compared to about 2–10% on agar plates in this study.

We also evaluated whether the NemaLife chip induces environmental stresses such as starvation in the animals. We chose a strain that expresses the stress reporter DAF-16::GFP^63^, which exhibits DAF-16 nuclear localization under caloric restriction, heat, and oxidative stresses^64–66^. We first determined that the strain harboring this reporter (TJ356) exhibited similar lifespan on both agar plates and in the microfluidic device, indicating that culture in the liquid environment of the microfluidic pillar device does not induce deleterious effects on TJ356 survival (Fig. 7b, see additional trial data in SI Fig. S2).

We then performed fluorescence imaging to assess DAF-16 localization. In the device at 20 °C, day 3 and day 8 animals, or on agar at day 8, animals did not exhibit such DAF-16::GFP localization (images in Fig. 7c-i, ii, iii). We quantified the visible puncta in day 8 animals cultured on agar plates and in microfluidic chambers and found no significant difference (Fig. 7c-iv). Thus, although animals cultured in our microfluidic device can induce stress responses similar to those cultured on agar, the standard growth conditions we use do not elicit *daf-16*-dependent translocation to the nucleus.

Over the course of the study, we conducted 20 separate lifespan assays of WT worms in the NemaLife chip (see SI Fig. S3), allowing us to account for seasonal changes in laboratory environments. We found that variation in lifespan is limited to 13%, a level of replicate variation comparable to that found in lifespan assays conducted in LSM technolology^62^.

#### Lifespan studies using mutants and RNAi interventions

To further test our NemaLife device, we sought to replicate phenotypes of well characterized long-lived and short-lived mutants. As a starting point, we chose established long-lived genetic mutants: insulin signaling mutants *daf-2(e1370)* (insulin receptor reduction of function mutation^67^)*, age-1(hx546)* (phosphatidylinositol-3-OH (PI3) kinase reduction of function mutation^68^) and eating-impaired dietary restriction mutant *eat-2(ad1116)*^69^. We also tested the short-lived, insulin signaling mutant, *daf-16(mgDf50)*, which lacks the FOXO transcription factor homolog^70,71^.

**Figure 8:**
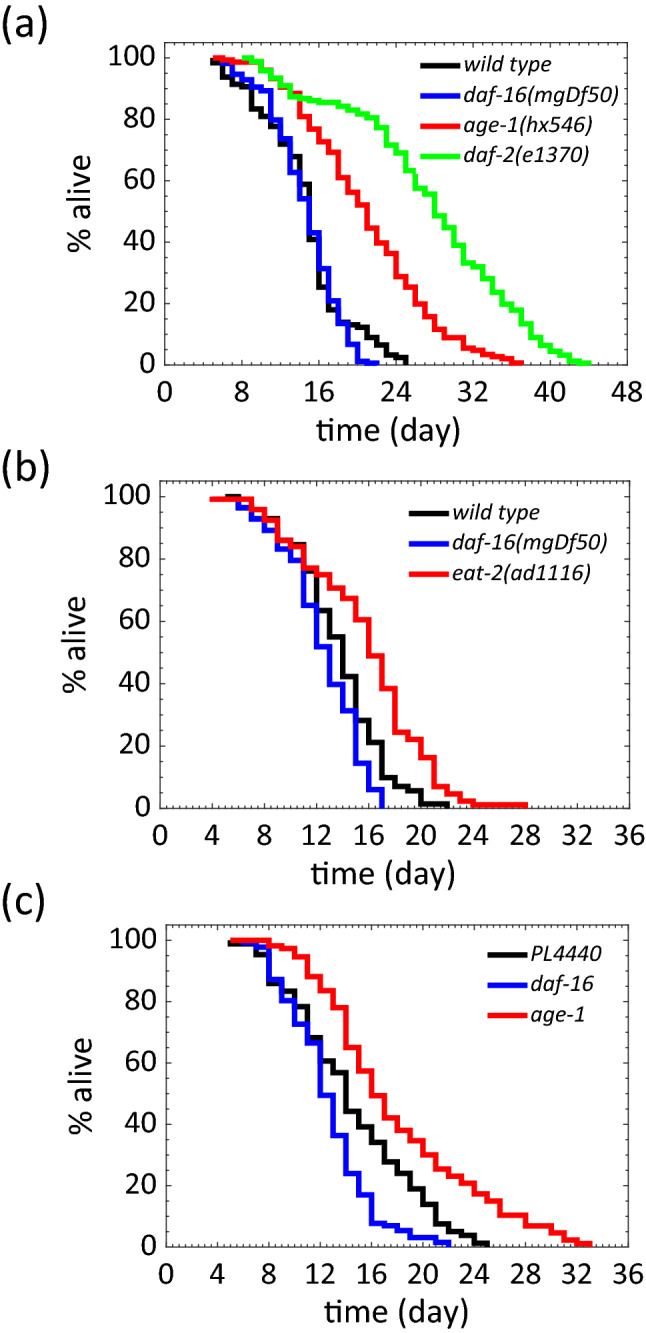
Mutant and RNAi testing in the NemaLife device. (**a**) Lifespan of long-lived mutants *daf-16(mgDf50), daf-2(e1370)* and *age-1(hx546).* N = 2 trials, data is shown for one trial. n = 160, 152, 168, 128 for *daf-2*, *age-1*, *daf-16* and wild type animals respectively. (**b**) Lifespan extension of *eat-2(ad1116)* establishes NemaLife chip for its suitability to carry out dietary restriction experiments. N = 1 trial. n = 123, 84, 86 for *eat-2*, *daf-16* and wild type animals respectively. (**c**) RNAi efficacy establishes the ability of the lifespan device to capture the on-chip genetic modification. For RNAi efficacy fragments targeting *daf-16* and *age-1* were inserted into feeding vector L4440. Wild type *C. elegans* of day 3 were used in the experiment. N = 3 trials, data is shown for one trial. n = 88, 135, 116 for *PL4440*, *daf-16* and *age-1* animals respectively. Food: 100 mg *E. coli* OP50/mL in S complete, feeding frequency: once/day.

Consistent with previous reports, *daf-2, eat-2* and *age-1* mutants exhibited robust extension of lifespan (Fig. 8a, b). For this data set, *daf-2*, *age-1*, *eat-2*, and wild-type shows a 100%, 64.2%, and 15.4% increase in median lifespan in the microfluidic pillar environment, respectively (Fig. 8a, b). For *daf-16* mutants, in Fig. 8a we did not observe a statistically significant difference, but we observed a significant decline in the trial shown in Fig. 8b and an additional trial shown in Table S2.

We note that the maximum lifespan of *daf-2* is 44 days, which demonstrates that the microfluidic culture environment can adequately support long duration longevity studies in *C. elegans*. We have also successfully measured lifespan of mutants with locomotory defects in the NemaLife chip indicating that fluid stress due to daily washing/feeding and our death scoring protocol does not elicit undesirable outcomes (Fig. S4). We conclude that NemaLife chip can both support long term culture of *C. elegans* and yield longevity outcomes that parallel those reported on agar plates.

We also tested whether genetic screens using RNAi can be pursued in the NemaLife device. In *C. elegans* targeted post-transcriptional RNA knockdown can be achieved by feeding animals bacteria that harbor clones expressing specific double stranded RNAs^72,73^. We found a 15.4% extension and a 15.4% reduction in median lifespan of worms fed RNAi targeting *age-1* and *daf-16,* respectively*,* when compared to worms fed an empty vector (PL4440) as a control (Fig. 8c). Likewise in terms of maximum lifespan, we found a 25% extension and a 8% reduction in *age-1* and *daf-16* respectively. RNAi efficacy in the NemaLife chambers establishes that food adequate to elicit the RNAi-mediated silencing of the targeted gene is ingested by the animal, addressing a potential concern on the efficacy of RNAi intervention in the microfluidic environment.

### Scoring healthspan measures in *C. elegans*

In addition to lifespan measurement, the transparency and shallow depth of the PDMS worm-habitat chamber offer the opportunity to evaluate physiological characteristics and fluorescent biomarkers of healthspan in *C. elegans*. In this study, we focused on manual scoring of pharyngeal pumping and stimulus-induced forward and reversal speed. The ability to score such phenotypes across lifespan demonstrates the capacity of NemaLife as a device for healthy aging investigations in *C. elegans*.

#### Pharyngeal pumping

The pharynx of *C. elegans* is a heart-like organ that uses rhythmic contraction and relaxation to facilitate bacterial uptake^74^. Pharyngeal pumping rates depend on several factors, such as food availability and environmental quality, and significantly decline with age, making pumping evaluation an attractive physiological marker for evaluating *C. elegans* health status^45,47^.

Lockery et al.^46^ developed a microfluidic device to measure pumping rates by recording the electrical activity of the pharynx while the animal is immobilized. Scholz et al. reported an image-based pharyngeal pumping measurement technique in which the movement of the grinder of an immobilized worm in a microfluidic device (WormSpa^75^) is tracked^76^. However, in both cases, the microfluidic devices are specifically designed to immobilize animals of a given body size, and as a result these approaches are not conducive for whole-life culture and recording pharyngeal pumping measurements across the lifespan of the animal. Likewise, in prior pillar-less microfluidic devices in which animals swim continuously, it is difficult to record pharyngeal pumping due to 3D motion.

**Figure 9:**
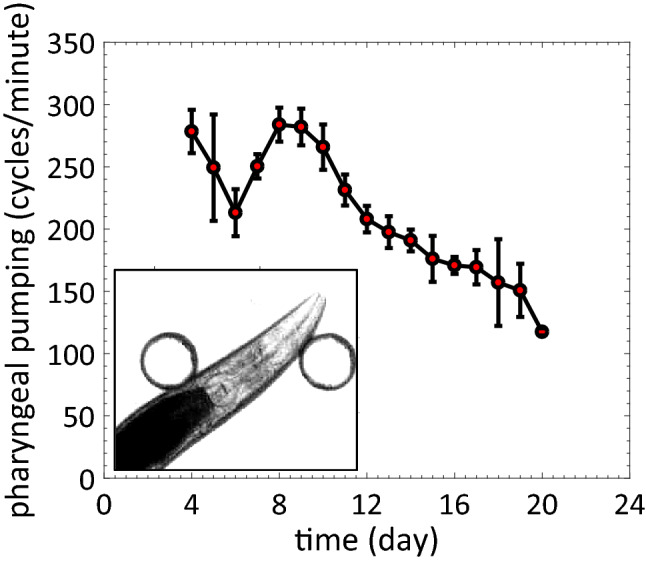
Age-associated decline in pharyngeal pumping rate as measured in the NemaLife chip. Pharyngeal pumping in cycles per minute for wild type *C. elegans* over the course of its lifespan (n = 10 at each time point). Error bar is the standard deviation. Food: 100 mg *E. coli* OP50/mL S complete, feeding frequency: once/day. Inset shows the pharynx of an animal inside a chamber.

Using our NemaLife platform, we confirmed previous reports of age-dependent reduction of pharyngeal pumping in wild-type *C. elegans* (Fig. 9, see Movies S4, S5). Additionally, we observe temporal changes in pharyngeal pumping rates throughout various life stages. In animals maintained within NemaLife, we report that pharyngeal pumping rates increase up to the end of the reproductive period, reaching a maximum of ≈ 283 cycles/min on day 8. Pumping rates decrease gradually starting from day 10, reaching ≈ 117 cycles/min on day 20, at which point, degeneration of the pharynx makes it difficult to score pumping frequency.

Maximum pharyngeal pumping rates we observed are similar to that reported on agar^45^, however, the rate of age-dependent decline in the pharyngeal pumping was relatively slow in the microfluidic device compared to agar. Old animals (day 15) grown on agar exhibited a significant reduction in pharyngeal pumping rate (20–30 cycles/min^45,47^) while animals housed in the microfluidic device maintain a relatively high pharyngeal pumping rate (150–160 cycles/min) in late life. Studies show that *C. elegans* increases rate of pharyngeal pumping with food concentrations in liquid environments^76–78^. It is possible that the abundance of high-quality food even after reproduction makes the decline less pronounced. It is also possible that less bacterial biofilm is formed in the microfluidic environment and therefore animals are less prone to bacterial colonization in the pharynx. Nevertheless, it is clear that the age-associated pumping rate decline can be monitored using the NemaLife chip. The high pharyngeal pumping rate in the NemaLife chip may be advantageous for enhanced uptake of compounds in pharmacological assays.

#### Stimulated locomotion

Locomotory vigor is commonly used as a healthspan measure in *C. elegans*^45,47,79^. Forward crawling of *C. elegans* is accompanied by pauses and reversals at a speed and frequency that are dependent upon food availability and the crawling environment. As a result, temporal fluctuations in locomotion make it difficult to evaluate true crawling speed. Extended tracking and long-term analysis of time-lapse images are required to properly assess forward locomotion dynamics. Reversals are observed during natural locomotion and are important for escape in response to gentle touch, a behavior in which the worm quickly reverses and suppresses head movement^80–83^. Spontaneous reversals are usually short episodes between consecutive forward crawling bouts. Reversal behavior is thus an indicator of neuro-muscular function^82^ that can be scored reliably in a short observation time.

On agar plates, reversals are induced by applying gentle touch to the animal with an eyelash or by prodding the worm with a platinum pick or by simply tapping the plate^80,84^. Here, we replicate the gentle touch stimulus in the microfluidic device with a hex key to induce stimulated reversals. We induced reversals mechanically by gentle tapping on the top surface of the device 3 times at a location slightly away from the worm pharynx. Stimulus is transferred to the worm as mechanical vibration through the pillar and the fluid. This stimulus-induced locomotory response cannot be evaluated in prior pillar-less microfluidic devices because it is difficult for the animals to exhibit reversals in a fully liquid environment. Thus, the tap-induced reversal is unique to the NemaLife chip.

**Figure 10:**
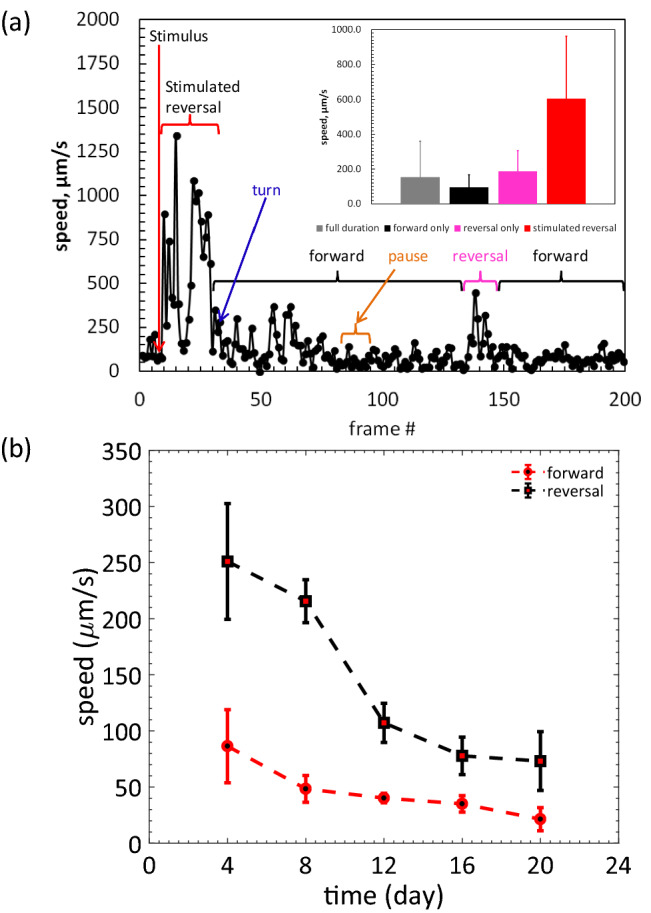
Stimulated reversal speed and age-associated locomotory decline in wild-type *C. elegans*. (**a**) A representative locomotion episode of a day-5 *C. elegans* over 40 s duration. Each filled circle represents instantaneous speed between two consecutive frames determined by manually tracking the vulva of the animal. Tap stimulus was applied (frame # 10, red arrow) to induce reversal. Animal executed reversal with a very high speed followed by second reversal. Animal turns (blue arrow), crawls in forward direction (black brackets) for relatively long time with pause (amber bracket). Naturally occurring reversal period is shown by the pink bracket. Average speed of the full episode, only forward (including pauses), only reversal and stimulated reversal are shown in the inset. Error bars are standard deviations. n = 5. (**b**) Decline in forward and first reversal speed as wild type *C. elegans* ages in the micropillar arena. Error bar is the standard deviation. n > 10, food: 100 mg *E. coli* OP50/mL S complete, feeding frequency: once/day.

Stimulated responses generally involve an initial reversal (first reversal), a change in direction, followed by a final forward movement (Fig. 10a). In nearly all cases, we observed that animals immediately respond to a tap by exhibiting a first reversal followed by a turn. Occasionally, we observed brief interruptions in forward crawling motion that appeared to be independent of the stimulus. Inset of Fig. 10a shows the average speed calculated from the different modes of crawling. As expected, the first reversal showed the highest speed.

Using the NemaLife chip, we show that both reversal and forward speed vary significantly with age and we find that reversal speed is always greater than forward speed in worms of all ages (Fig. 10b). Most importantly, the rate of decline in reversal speed is accelerated at the end of reproduction. More specifically, between day 8 and day 12, the reversal speed measure is 50% reduced, whereas stimulated forward speed is only 17% reduced. Interestingly, the decline in the stimulated reversal speed is correlated with an equally rapid decline in survival rates following the reproductive period, an observation that underscores the importance of expanding lifespan assays to include the evaluation of additional health span metrics. Overall, the NemaLife chip enables measurement of stimulated reversal speed as a novel biomarker for aging and healthspan, which couples with other health measures to establish a powerful platform for analysis of *C. elegan*s healthspan and lifespan.

### Assessment of throughput of NemaLife chip

In this study, we developed NemaLife chip for life-long culture of *C. elegans* focusing on lifespan assays. Standard lifespan assays on NGM plates involve manual steps of animal transfers and scoring animal deaths. The operational protocol for NemaLife chips also involves manual steps of washing, feeding and scoring for live/dead animals. Therefore, it is useful to evaluate the throughput of conducting lifespan assays with the NemaLife chip.

To estimate the labor time taken for conducting a lifespan assay with NemaLife chip and plates, we considered a sample size of 100 animals and the maximum lifespan as 4 weeks. This sample size approximately corresponds to one NemaLife chip (9 chambers × 10–15 animals per chamber) or 4 NGM plates (4 plates × 30 animals per plate). Table 2 shows the estimated labor time when the assay is conducted on NGM plates or NemaLife chip.

**Table 2:** Estimates of labor time needed for conducting a lifespan assay on NGM plates and NemaLife chip.

	NGM plates		NemaLife chip		
Tasks	Animal transfers	Scoring live/dead	Progeny washing	Animal feeding	Scoring live/dead
Daily labor time	20–40 min (5–10 min per plate × 4 plates)	12–20 min (3–5 min per plate × 4 plates)	9 min (1 min per chamber × 9 chambers)	3 min (20 s per chamber × 9 chambers)	5 min (0.5 min per chamber × 9 chambers)
Total person hours	15—28 h (32–60 min per day × 28 days)		7.9 h (17 min per day × 28 days)		

The sample size for the estimation is taken to be 100 animals and the maximum lifespan as 4 weeks. Note that for lifespan assays in both NGM plates and the Nemalife Chip, the scoring time decreases in later days as animals die.

Overall, we find that a lifespan assay takes about 2X–4X less labor time with NemaLife chip than on plates. The significant savings in time come from eliminating manual picking of animals as well as from scoring. In the NemaLife chip, progeny washing is done by a hand-syringe (connected with a tubing and a pin) allowing the user to wash one chamber and move to the next. The scoring in NemaLife chip also takes less time since the population of 100 animals is compartmentalized into 9 smaller chambers with fewer animals compared to plates. On NGM plates, animals explore a larger footprint and with a limited field of view which requires searching. In addition, death events can be problematic to score in translucent agar, whereas on the chip the transparency of PDMS, narrow chamber height and cleaner background reduces the time required to score.

In Table 2, we have only listed the labor time for conducting the assay, but we did not consider the preparation time which includes making plates or chips, obtaining synchronized animals, growing bacteria and storing plates or chips in incubators. The preparation time for animal and bacteria culture are similar for the two approaches, however, the preparation time for NGM plates may be longer than the NemaLife chips since a single chip is required for a full lifespan assay while 15–20 NGM plates are required depending on the frequency of animal transfers.

In general, other considerations such as device failure, contamination, and clogging can affect the throughput of NemaLife chip. During the course of this study, we used 33 chips for lifespan assays and only 11 out of 247 chambers (4.45% chambers) failed during the experiment. The failure is due to human errors such as forgetting to block the worm loading port with a solid pin which causes animals to escape during washing/feeding, or adding ethanol accidentally in one chamber. Despite these inadvertent errors, we were still able to obtain experimental data since the chip has 9 chambers and censoring of one or two chambers provides meaningful data although at a reduced sample size. We did not observe any major failure of NemaLife chambers due to clogging. The NemaLife chambers are designed symmetrically with respect to inlet and outlet. Our protocol involved using each port alternatively and thus keeping the sieve channels free of clogging. In addition, contamination of chips was not observed due to pre-sterilization of chambers prior to experimentation and also because the enclosed chamber minimizes exposure of the small amount of culture fluid (≈ 6.75 μL) to ambient environment. Finally, we note that the NemaLife chips cannot be reused for lifespan as the chambers accumulate chemicals and bacterial residue over the lifespan assay potentially influencing aging biology and lifespan of *C. elegans* if chips are re-used.

## Conclusions

We demonstrated that *C. elegans* can be effectively maintained in our NemaLife microfluidic device across its lifespan without using chemicals (progeny-blocking drugs, antibacterial agents, antifungal compounds, etc.) in an environment that recapitulates longevity on agar plates. The closed microfluidic environment keeps animals inside the arena boundary and eliminates the events of death by desiccation. Being fully occupied with fluid, the culture environment maintains uniform humidity and minimizes temperature fluctuations. Micropillars in the microfluidic device enable the animals to maintain natural crawling gaits and eliminate swim-induced stress. The well characterized survival outcomes of “cornerstone” longevity mutants with altered insulin-like signaling or dietary restriction pathways grown on agar are reproduced in the device; RNAi can also be executed.

The capacity for manual injections of fluids and food enables investigator control over feeding conditions or introduced drugs. For compound testing, the liquid environment in the NemaLife chip ensures the uniform availability of the compound which is difficult to ensure in agar-filled plates. In the NemaLife chip, we replace the old environment (larva, eggs, 24 h old bacteria and buffer, pheromones, excrete etc.) daily with fresh food. As a result, animals can be exposed to a fresh dose of compounds daily, similar to dosing schedules with mammalian models. An added benefit of the device is that progeny and effluent (containing any chemicals such as, pheromones etc.) might be collected for downstream analysis.

The NemaLife chip was also designed to improve the scoring of live/dead animals. The microfluidic chip consists of 9 smaller chambers on a 2" × 3" glass slide. Small footprint of an individual chamber is entirely visible at 10 × or 20 × magnification on a stereomicroscope. Each chamber was optimized to house 10–15 animals so that one can score animal surival easily. Thus, we achieve a large enough sample size for drawing meaningful statistical inferences without employing a computer vision system and complicated imaging system otherwise required for a larger chamber. These considerations make the NemaLife chip easily implementable in research labs conducting lifespan assays and investigator scoring options provide quality control on data.

In sum, the NemaLife chip is a simple and low-cost means to obtain reliable lifespan and physiological data on aging animals. The NemaLife chip can be developed into a fully automated platform, that can offer progeny removal, feeding, drug delivery, and phenotypic scoring using automated pump systems and an appropriate software. Thus, the NemaLife chip has a significant potential both as a low-cost “manual” device that can be used in many labs and as a core component of a fully automated platform.

## Experimental procedures

### Worm culture

All animals were cultured on 60 mm petri dishes containing nematode growth medium (NGM) at 20 °C before loading into the microfluidic chamber. The NGM filled petri dishes were seeded with 300–400 μL of bacteria *Escherichia coli* OP50 and incubated for 48 h at 20 °C. For age synchronization, 20–25 gravid adults were placed on seeded plates to lay eggs for 2–4 h. After eggs were laid, the animals were removed from the plates, eggs were incubated for 60–72 h. The day the eggs were laid was scored as day 0. In this study, we used wild-type Bristol (N2), GR1307[*daf-16 (mgDf50)I*]*, CB1370[daf-2 (e1370)III*]*,* TJ1052[*age-1(hx546)II*], DA1116[*eat-2(ad1116)II*] and TJ356[*zIs356* [*daf-16*p::*daf-16a/b*::GFP + *rol-6(su1006)]IV*]. Wild-type (N2), *daf-16,* and *daf-2* mutants were received from the *Caenorhabditis* Genetics Center (CGC); *age-1* and *eat-2* strains were kindly provided by the Driscoll lab.

### Device fabrication and preparation

All microfluidic devices were fabricated in poly(dimethyl)siloxane (PDMS) using soft lithography^85^. A mold was fabricated using two-step SU-8 photolithography such that the chamber height is ≈ 100 μm and the micropillar height is ≈ 75 μm, as described previously^52^. A 4–6 mm thick PDMS (Sylgard 184 A and B, 1:10 by weight, Dow Corning) layer was casted on to the mold and the inlet/outlet holes were punched with a 1 mm hole puncher. The PDMS device was then bonded on a glass surface irreversibly and rendered hydrophilic by plasma treatment (Harrick Plasma Inc.). Before using the device for lifespan experiments, the device interiors were filled with 70% ethanol for 5 min to sterilize them. Subsequently, the device was rinsed 4–5 times with S-complete solution. Devices were then treated with 5 wt% Pluronic F127 (Sigma-Aldrich) for 30 min to prevent protein and bacterial build-up^36^. In addition, Pluronic treatment also assists with removal of air bubbles if any are trapped. After incubation, excess Pluronic was removed by washing with S-complete. The Pluronic-treated devices were stored in moist petri dishes at 20 °C for immediate use and at 4 °C for future use.

### Food preparation

*E. coli* OP50 was used as the bacterial food source for worms grown on both NGM and maintained within the devices. Bacterial suspension of 100 mg mL^−1^ in S-complete solution corresponding to ~ 2 × 10^11^ was used for lifespan assays unless otherwise noted. E. *coli OP50* was grown overnight at 37 °C in standard LB broth. Bacterial suspensions of 100 mg mL^−1^ were prepared by centrifuging 500 mL of overnight bacterial culture and resuspending the pellet in S-complete. Concentrated OP50 was stored at 4 °C for subsequent use, up to 2 weeks.

### Bacteria preparation for RNAi studies

Engineered bacteria expressing double-stranded RNA (dsRNA) were obtained from the Driscoll Lab and used for testing the RNAi efficacy in the device. Fragments designed for targeting *daf-16* and *age-1* were cloned into the L4440 feeding vector and the resulting plasmids were transformed into the HT115 (DE3) using standard protocols^73^. Bacteria with empty L4440 vector (no cloned fragments) were used as the negative control for all experiments involving RNAi. Bacterial colonies were grown on LB agar supplemented with 50 µg/ml carbenicillin for 48 h at 37 °C. Fresh plates were made each week.

Single colonies of bacteria were picked and grown in culture flasks with shaking at 200 rpm for 16 h in sterile LB broth with 50 µg/ml ampicillin at 37 °C. For induction, 0.4 mM Isopropyl β-D-1-thiogalactopyranoside (IPTG) was added for 2 h at 37 °C while shaking. At 18 h, additional IPTG was added for a final concentration of 1 mM. Concentrated bacterial solutions were prepared each day using procedures described above. Final IPTG concentration was maintained at 1 mM in the food solution.

### Bacterial density measurement and food consumption

To assess whether the diet of the animals becomes restricted during overnight incubation, we determined the amount of food consumed by the animals during reproduction, a period of peak food consumption. We first obtained a calibration curve relating bacterial density in cfu/mL to optical density readouts. Using this calibration, we determined the daily consumption of food by the animals. A Synergy HT plate reader was calibrated for *E. coli* OP50 culture using standard microbiological techniques. Briefly, following the growth of overnight culture at 37 °C, 100 mg/mL *E. coli* OP50 in S-medium was serially diluted to 2^11^. Density of diluted suspensions was determined by measuring the absorbance of 150 µL aliquots in a flat-bottom 96 well plate. Each diluted suspension was subsequently serially diluted to 10^–7^ and 0.1 mL of bacteria was plated onto standard LB plates. Plates were incubated at 37 °C, and individual colonies were counted after 24 h. A plot of cfu/mL against OD600 readings of the original sample was plotted to obtain a linear regression.

9–14 Young adult (65 h) worms were loaded into NemaLife housing chambers by using 1 mL syringe. 100 µL of *E. coli* OP50 suspension (100 mg/mL) was pushed through the chamber to eliminate potential dilution by the liquid buffer (~ 6.75 µL) that remains in the chamber. To determine the concentration of bacterial cells upon feeding each chamber was washed with buffer and the solution collected immediately after introducing bacteria to the chamber. Microfluidic chips were stored at 20 °C for 24 h, then chambers were washed with buffer and fluid is collected for each. Fresh *E. coli* OP50 is then replaced as described above. Each sample from before and after incubation is centrifuged at 3500 RPM for 10 min and resuspended in 1 mL of fresh buffer. Bacterial density (cfu/mL) was determined by measuring the optical density (wavelength 600 nm) of 150 µL of the fluid collected from the chamber and resuspended in buffer. OD600 measurements are applied to our calibration curves (see below). We determined an OD600 of ~ 1.0 correlates to 4.1 × 10^11^ cells/mL.

### Fluorescence imaging

We imaged the *C. elegans* strain containing stress reporter gene zls356IV inside the device without immobilizing them using a Nikon Ti microscope at 10X magnification. Movies were captured using fast time lapse imaging with a camera (Zyla 5.5 sCMOS from Andor Inc.). For imaging worms cultured on agar plates, worms were loaded into a fresh chamber and imaged immediately. Movies were analyzed using ImageJ (NIH) software.

### Scoring animal death

Worms were counted manually, and lifespan was scored daily. An animal was scored as dead if it failed to respond to (1) gentle flow of fluid throughout the chamber or (2) gentle tapping of the device by a 3/8″ Allen key. If there is no movement in the pharynx or in the tail 1 min after the stimulus has been applied, we scored the animal as dead. Each death event was scored as 1 and unaccounted deaths (missing, washing error, matricides) were scored as 0. A lifespan curve (Kaplan–Meier) was then generated. Kaplan–Meier curves and Log-Rank statistics were generated using the Statistics Toolbox in MATLAB.

### Locomotory measures

Reversal and forward speed were scored after a gentle tap on the PDMS device with a 3/8″ Allen key, at a location close to the tip of the pharynx. Only the initial, instantaneous reversal following stimulation were scored. Continuous frames of a spontaneous start of reversal and end of reversal were taken as a complete reversal episode. Reversal episodes with a pause, stop, or intermittent reversal were not included. Continuous forward locomotion until a spontaneous reversal was scored to determine forward speed. Images were captured using SVSi streamview camera at 10 frames/s. Locomotion speed was calculated by tracking the displacement of the pharynx in time. The maximum speed in an episode (forward/reversal) of a worm was recorded as the maximum reversal/forward speed.

### Pharyngeal pumping

Movies of 10 individual worms were captured at a rate of 20 frames/s with SVSi streamview camera on a Zeiss stereo microscope at 5 × magnification, 15 min after the addition of food to the device. Complete cycle time of contraction/retraction of the isthmus/terminal bulb of pharynx was manually computed using ImageJ (NIH). The number of pharyngeal pumping cycles were counted over a 10 s period for each single animal and then reported as cycles per minute.

### Data analysis

All the survival analyses were conducted in MATLAB (Mathworks, R2014b). Log-rank (Mantel-Cox) test was used to compare survival between treatment groups. Two-sample *t* test was used to compare growth of the worm and crawling kinematics in the optimization study.

## Supplementary information

Supplementary Information.

Supplementary Movie 1.

Supplementary Movie 2.

Supplementary Movie 3.

Supplementary Movie 4.

Supplementary Movie 5.
