# Supplementary material for: NemaLife chip: a micropillar-based microfluidic culture device optimized for aging studies in crawling C. elegans
Source: Sci Rep. 2020 Oct 1;10:16190. doi: 10.1038/s41598-020-73002-6 (PMC7530743; doi:10.1038/s41598-020-73002-6)

**NemaLife Chip: A micropillar-based microfluidic culture device optimized for aging studies in crawling *C. elegans***

Mizanur Rahman^1^, Hunter Edwards^1^, Nikolajs Birze^1^, Rebecca Gabrilska^2^, Kendra P. Rumbaugh^2^, Jerzy Blawzdziewicz^3^, Nathaniel J. Szewczyk^4^, Monica Driscoll^5^ and Siva A. Vanapalli*^1^

^1^*Department of Chemical Engineering, Texas Tech University, Lubbock, TX 79409, USA.*

*^2^Department of Surgery, Texas Tech University Health Sciences Center, Lubbock, TX 79409, USA.*

*^3^Department of Mechanical Engineering, Texas Tech University, Lubbock, TX 79409, USA.*

*^4^Ohio Musculoskeletal and Neurological Institute and Department of Biomedical Sciences, Ohio University, Athens, OH, 45701.*

*^5^Department of Molecular Biology and Biochemistry, Rutgers University, Piscataway, NJ 08854, USA.*

**Supplementary Information (SI)**

Supplementary Materials is uploaded as a separate file. List of the items are

Fig S1: Changes in body size of *C. elegans* cultured in the NemaLife device.

Fig S2. Lifespan replicates of a transgenic strain TJ356 with a Pdaf-16::GFP stress reporter on agar plates and in the microfluidic device.

Fig S3. Natural variation in lifespan of wild-type *C. elegans* scored in NemaLife device

Fig S4. Lifespan of *unc-52(e669)* and *unc-112* *(r367ts)V* mutant scored in microfluidic chambers.

Table S1: Measured dimensions of the micropillar geometries used in the study for device optimization.

Table S2. Summary of the lifespan trials conducted in the study

**Movie S1: Microfluidic habitat chamber with adults and progeny**.

**Movie S2: Progeny washing in pillar laden microfluidic chamber.** Pillars in microfluidic habitat chamber allow adults crawl but facilitate progenies flow with buffer between pillars. Sieve channels retain the adults which are near the exit.

**Movie S3: Progeny washing in pillar-less microfluidic chamber.** Adult animals flow with the buffer while washing, accumulate at the sieve channel and blocks fluid flow.

**Movie S4: Pharyngeal pumping in day 1 adult in the NemaLife chip in the presence of food.** The frame rate is 20 fps.

**Movie S5: Pharyngeal pumping in day 10 adult in the NemaLife chip in the presence of food.** The frame rate is 20 fps.

* * *

* * *

(a)

(b)

* * *

* * *

*

**Fig S1: Changes in body size of *C. elegans* cultured in the NemaLife device.** (a) Average worm length of wild type *C. elegans*. Error bars are standard deviations. n>10. (b) Average worm diameter of wild type *C. elegans*. Error bars are standard deviations. n>10, two sample t-test, *P-value (once/day and once every other day) < 0.01, *** P-value (once/day and once every other day) < 0.0001.


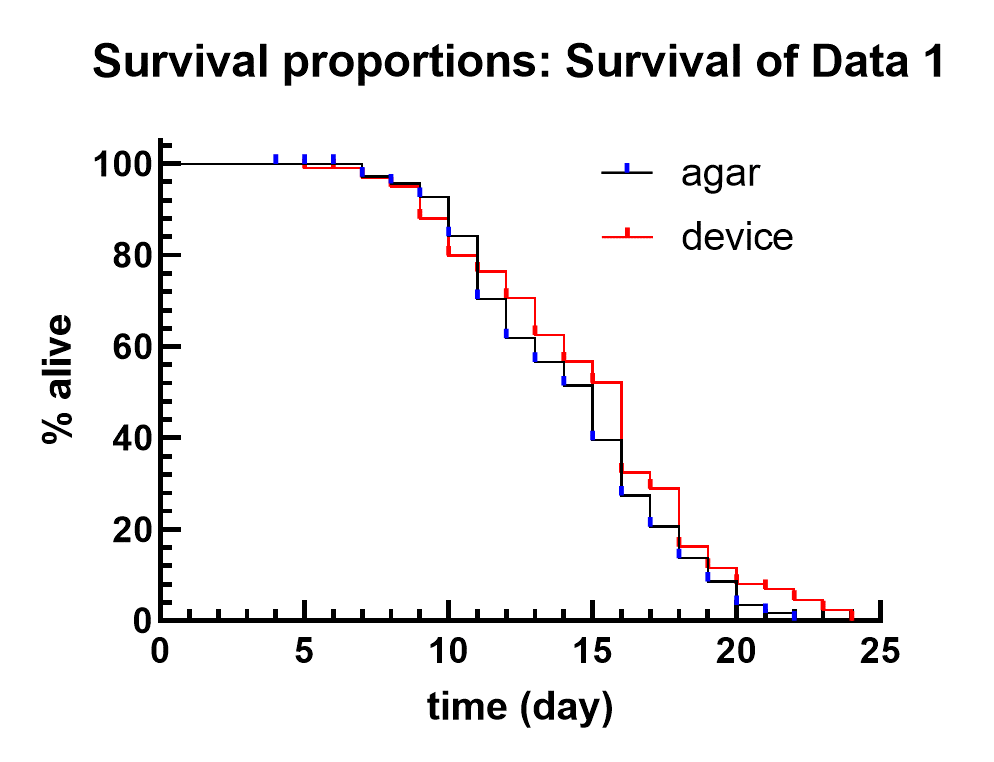


**Figure S2**. Lifespan replicates of a transgenic strain TJ356 with a Pdaf-16::GFP stress reporter on agar plates and in the microfluidic device (p=0.45, log rank test). Sample size is 91 and 103 animals for agar and NemaLife chip. Trials conducted at 20℃.

**Fig S3. Natural variation in lifespan of wild-type *C. elegans* scored in NemaLife device.** Lifespan of 20 trials of wild-type animals cultured and scored in NemaLife. Experiments were conducted randomly at different seasons in laboratory environments. Thin black lines are the lifespan curves for individual trials, thick red line represents the average of the lifespans derived from the set of 20 experiments. Mean lifespan (95 % C.I.) 13.4 – 15.2 days and maximum lifespan 21.6 – 24.3 days (Range of median lifespan 11 – 19 days and maximum lifespan 17 – 27 days). n = 60 - 150 animals.


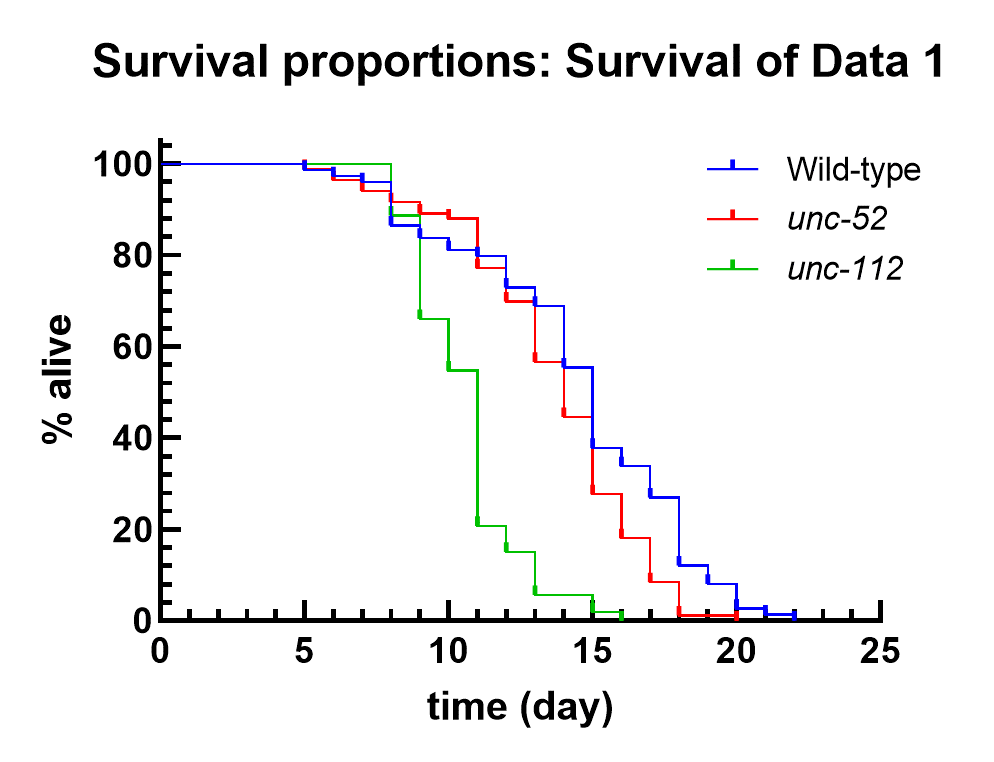


**Figure S4**. Lifespan of *unc-52(e669)* and *unc-112* *(r367ts)V* mutant scored in microfluidic chambers (p <0.0001, log rank test). Median lifespan is 15, 14, and 11 days respectively for wild-type, *unc-52*, and *unc-112* (maximum lifespan 22, 20, and 16 days respectively). Sample size is 74, 83 and 95 animals for wild-type, *unc-52*, and *unc-112* respectively. Trials conducted at 20℃.

**Table S1: Measured dimensions of the micropillar geometries used in the study for device optimization.**

**Table S2. Summary of the lifespan trials conducted in the study.**


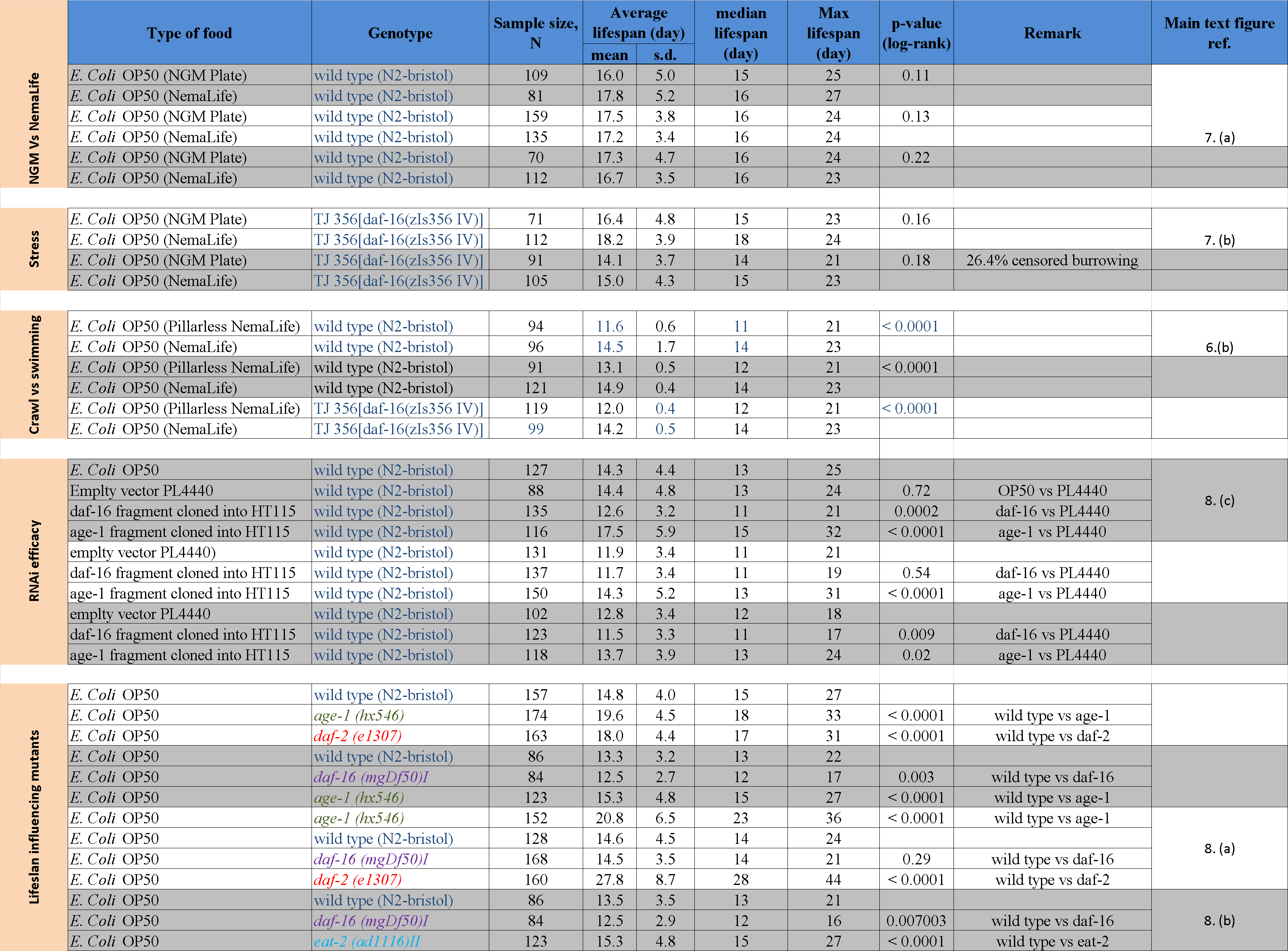

Supplement: Supplementary file 1 — Supplementary Information. [file 41598_2020_73002_MOESM1_ESM.docx]
